# Knowledge regarding human papillomavirus and cervical cancer prevention among medical students from Chulalongkorn University in Thailand

**DOI:** 10.1186/s12905-024-02933-3

**Published:** 2024-02-19

**Authors:** Monchada Sukrong, Peerapong Prapaisilp, Tunchanok Juntamongkol, Noppachai Siranart, Natacha Phoolcharoen, Nicha Assavapokee, Nakarin Sirisabya, Somsook Santibenchakul

**Affiliations:** 1 Faculty of Medicine, Chulalongkorn University, 1873 Rama IV Road, Pathumwan, Bangkok 10330 Thailand; 2 Department of Obstetrics and Gynecology, King Chulalongkorn Memorial Hospital, Faculty of Medicine, Chulalongkorn University, 1873 Rama IV Road, Pathumwan, Bangkok 10330 Thailand

**Keywords:** Human papillomavirus, Cervical cancer, Screening, Prevention, Knowledge, Medical student

## Abstract

**Background:**

Cervical cancer is one of the leading causes of death among women in Thailand. General practitioners, within their primary healthcare role, play a vital role in the cervical cancer screening program, as they are the healthcare professionals most easily accessible to the general population. This study aims to determine the level of knowledge of cervical cancer and human papillomavirus (HPV) infection, HPV vaccination, and cervical cancer screening among last-year medical students.

**Methods:**

A cross-sectional study was conducted among sixth-year medical students using an electronic self-administered questionnaire. The two-part questionnaire comprised demographic data and 12 true/false questions that assessed knowledge regarding HPV infection, HPV vaccination, and cervical cancer screening recommendations. Pilot testing revealed a high Cronbach’s alpha and test–retest reliability coefficient.

**Results:**

A 67% response rate was achieved. Among the 198 respondents, only one (0.5%) student correctly answered over 80% of the questions while most respondents (172, 71.7%) correctly answered less than 60% of the questions. Less than half of the respondents correctly identified crucial aspects such as the primary cause of cervical cancer, recommended vaccination age, cytology sensitivity compared to HPV testing, and the recommended screening frequency for average-risk women.

**Conclusions:**

This study highlights a significant lack of comprehension among Thai medical students concerning HPV infection, vaccination, and cervical cancer screening guidelines. Encouraging educational enhancement, effective communication, and heightened awareness of these crucial topics within the medical school curriculum are imperative.

## Introduction

Cervical cancer is a leading cause of death worldwide despite the availability of effective prevention interventions for over seven decades [1]. In recognition of the morbidity and mortality associated with this preventable disease The Global Strategy to accelerate the elimination of cervical cancer as a public health problem 2020–2030 has set goals for 2030 with a view to accelerate the progress to the elimination target of an incidence of 4 per 100 000 [2].

The Thailand National Cervical Cancer Program [3], established in 2005 by the Ministry of Public Health (MOPH), relies on Pap smear and Visual Inspection with Acetic Acid (VIA) methods for screening. Over time, this program has demonstrated remarkable success in reducing cervical cancer incidence rates. Beginning at 23.4 per 100,000 women in 1989, when cervical cancer was the most prevalent cancer among women, the rate declined to 11.7 per 100,000 women by 2014. Since 2017, the Thai government has endorsed HPV vaccination as a primary preventive measure. This initiative involves administering two doses of the bivalent HPV vaccine, spaced six months apart, specifically targeting grade 5 schoolgirls within the National Immunization Program. As of 2021, the mean annual Age-Standardized Rate (ASR) of cervical cancer in Thailand is reported at 11.3 per 100,000 women [4].

In Thailand, the majority of doctors operating within district hospitals—integral parts of the primary healthcare system—comprise general physicians. These healthcare professionals play a pivotal role in population-wide disease prevention efforts [5]. Following medical school graduation, most medical students are mandated to serve in provincial or district hospitals as general physicians.

Primary care physicians (PCPs) play a critical role in enhancing the effectiveness of both primary and secondary cancer prevention. Their impact extends beyond tobacco cessation to addressing various cancer risk factors outlined in the European Code Against Cancer (ECAC), such as obesity, unhealthy diet, lack of physical activity, alcohol consumption, and low engagement in screenings [6].

To our knowledge, no studies have assessed the knowledge of final-year medical students regarding cervical cancer prevention. This study aimed to thoroughly document the level of understanding concerning HPV and cervical cancer prevention among sixth-year medical students at Chulalongkorn University in Bangkok, Thailand.

## Materials and methods

### Study design

This study used a cross-sectional design. The authors distributed an electronic self-administered questionnaire to all sixth-year medical students at Chulalongkorn University in May 2021. Study data were collected and managed using Research Electronic Data Capture tools hosted at Chulalongkorn University [7]. Only consenting students were eligible to participate in the survey. The study was approved by the Institutional Review Board of the Faculty of Medicine at Chulalongkorn University.

### Questionnaire

The authors developed a two-part, self-administered questionnaire. In the first part of the questionnaire, demographic data were collected using eight questions on gender, age, rotation, and specialty of interest. The second part comprised 12 knowledge-based true/false questions including queries on the three HPV-based topics: (1) HPV infection and cervical cancer (three questions), (2) HPV vaccination (four questions), and (3) cervical cancer screening (five questions). The Twelve true/false questions with the answers were demonstrated on Table 1. Each question had three possible answers: true, false, and I don’t know. The investigators have provided the correct answers based on the fundamental knowledge according to the standard guidelines [8–10]. Face validity was assessed by two obstetrician-gynecologists (SS and NP).

**Table 1:** Twelve true/false questions with the answers

Question	Answer
1. Precancerous cervical lesions and cervical cancer are strongly associated with sexually transmitted high-risk HPV infection, which causes more than 97% of cervical cancers.	True
2. HPV types 16 and 18 are high-risk strains that cause around 70% of all cervical cancers.	True
3. The low-risk HPV (types 6 and 11) cause 90–95% of anogenital warts and 30% of cervical cancers.	False
4. HPV vaccine can reduce the risk of cancers include cervix, vaginal, vulvar, anal, penile, and oropharyngeal cancers.	True
5. Cytology is more sensitive than HPV testing in detecting CIN2 and CIN3.	False
6. Women with visible cervical lesions on speculum examination should undergo screening for cervical cancer.	False
7. For women age 30 to 65 years, cytology alone every three years is the preferred method of cervical cancer screening.	False
8. Annual screening for cervical cancer is not recommended for women with average risks at all ages.	True
9. Women who underwent total hysterectomy with removal of the cervix unrelated to cancer should continue to screen for 20 years with cytology every three years.	False
10. The Centers for Disease Control and Prevention recommended for HPV vaccination to include vaccinating boys and girls before 15 years of age, and as early as nine years of age.	True
11. A two-dose series of HPV vaccine is used when initiated before 12 years of age, whereas a three-dose series is required if initiated at 12 years or older.	False
12. If Cervarix (bivalent vaccine) or Gardasil (quadrivalent vaccine) has already been given, the patient should be revaccinated using Gardasil-9 due to more coverage of HPV genotypes.	False

### Questionnaire analysis

The questionnaire was pretested with 10 first-year obstetrics and gynecology residents to assess the clarity of the questions and was subsequently revised to match participants’ level of understanding. The internal consistency of the knowledge-based questions was evaluated; the Cronbach’s alpha of each of the three HPV-based topics was 0.8, 0.7, and 0.7. The test–retest reliability coefficient for all topics was 0.8.

### Recruitment

The survey was distributed in the closed online group chat for sixth-year medical students, a total of 296 students. Each participant received an anonymous electronic link to access the questionnaire.

### Outcome variables

The primary outcome was the knowledge score, which was based on the number of correct answers to the 12 knowledge-based true/false questions. A correct answer scored 1 whereas an incorrect answer or an “I don’t know” response scored 0. The lowest and highest possible knowledge scores were 0 and 12, respectively.

### Statistical analysis

The statistical analysis was performed using Statistical Package for Social Science, Version 27.0 for Mac (IBM Corp., Armonk, NY, USA). The categorical variables were presented by frequency and percentage and the numerical variables were presented by mean and standard deviation. The Kolmogorov–Smirnov test was employed to evaluate the normality of participants’ total scores, which were derived from their correct responses to the questions. Differences in average scores between sample subgroups were examined using independent t-tests and one-way analyses of variance. A *p*-value less than 0.05 was considered statistically significant.

## Results

### Baseline characteristics

From 296 students in the closed online group chat for sixth-year medical students, 198 students responded (66.9%). The average age of the 198 respondents was 23.1 ± 1.2 years. The number of males and females was equal (*n* = 96, 48.5% each). Six students (3%) described themselves as non-binary, gender-fluid, or agender or preferred not to divulge gender. Information on the duration between finishing the obstetrics and gynecology rotation and completing the questionnaire was collected, and respondents were classified into three groups. Among respondents, 44.4% (*n* = 88), 33.8% (*n* = 67), and 21.8% (*n* = 43) completed the questionnaire < 15 months, 15–18 months and > 18 months after finishing their obstetrics and gynecology rotation, respectively. Surgery or orthopedics (*n* = 42, 21.2%) was the most common intended specialty, followed by internal medicine or pediatrics (*n* = 41, 20.7%) and obstetrics and gynecology (*n* = 12, 6.1%); however, many students stated they had not yet decided on a specialty.

### Analysis of responses in three knowledge aspects in the questionnaire

Questions in the questionnaire were weighted equally to ensure the total score was 12. The mean knowledge score was 6.12 ± 1.90, with 0 and 11 being the lowest and highest scores, respectively, out of a total score of 12. The results of Kolmogorov–Smirnov test, with a p-value greater than 0.05, indicated that the knowledge scores were normally distributed. Table 2 presents the number and percentage of students who provided correct answers regarding three aspects of knowledge assessed in the questionnaire.

Two out of three items regarding HPV infection and cervical cancer were answered correctly by most respondents. However, less than one-third of the students (*n* = 59, 29.8%) possessed enough knowledge about low-risk HPV (types 6 and 11).

**Table 2:** Twelve knowledge-based assessments with the number of correct answers

Knowledge assessed from the questionnaire	Number of correct answers (%)
**HPV infection and cervical cancer**	
1. HPV types 16 and 18 are high-risk strains that cause approximately 70% of all cervical cancers.	184 (92.9)
2. Precancerous cervical lesions and cervical cancer are strongly associated with sexually transmitted high-risk HPV infection, which causes more than 97% of cervical cancers.	166 (83.8)
3. Low-risk HPV (types 6 and 11) causes 90 to 95% of anogenital warts but doesn’t cause cervical cancer.	59 (29.8)
**HPV vaccination**	
4. The HPV vaccine can reduce the risk of cancer including cervical, vaginal, vulvar, anal, penile, and oropharyngeal cancers.	136 (68.7)
5. The Centers for Disease Control and Prevention recommended HPV vaccination for boys and girls before 15 years of age and as early as 9 years of age.	104 (52.5)
6. If Cervarix (bivalent vaccine) or Gardasil (quadrivalent vaccine) has already been given, the patient doesn’t need to be revaccinated using Gardasil-9.	91 (46.0)
7. A two-dose series of HPV vaccine is used when initiating therapy before 15 years of age, whereas a three-dose series is required if initiating therapy at 15 years or older.	56 (28.3)
**Cervical cancer screening**	
8. Women who have undergone total hysterectomy unrelated to cancer can stop screening for cervical cancer.	111 (56.1)
9. For women aged 30 to 65 years, co-testing every 5 years is the preferred method of cervical cancer screening.	98 (49.5)
10. Annual screening for cervical cancer is not recommended for women of any age at average risk.	78 (39.4)
11. HPV testing is more sensitive than cytology in detecting CIN2 and CIN3.	77 (38.9)
12. Women with visible cervical lesions on speculum examination should undergo biopsy for cervical cancer.	52 (26.3)

*Abbreviation*: HPV, Human papillomavirus; CIN, cervical intraepithelial neoplasia

Among four items regarding HPV vaccination, two were correctly answered by more than half of the students. In contrast, only 46.0% (*n* = 91) and 28.3% (*n* = 56) of participants correctly responded to questions regarding revaccination and cut-off age, respectively.

The final part of the questionnaire specific to cervical cancer screening was the most difficult for participants; only one out of five items was correctly understood by approximately half (*n* = 111, 56.1%) of respondents. Questions regarding the preferred cervical cancer screening method for women aged 30 to 65 years, annual screening in average-risk women, HPV testing sensitivity, and the management of visible cervical lesions on speculum examination received correct responses from only 49.5% (*n* = 98), 39.4% (*n* = 78), 38.9% (*n* = 77), and 26.3% (*n* = 52) of respondents, respectively. Overall, respondents appeared to have a better understanding of the association between HPV infection and cervical cancer than of HPV vaccination or cervical cancer screening protocol.

### Factors affecting average scores

Female respondents (6.2 ± 1.8) received slightly higher average scores than male respondents (6.0 ± 2.1). The time gap between completing the obstetrics and gynecology rotation and answering the questionnaire appeared to have no effect; students who completed the department rotation 15–18 months before answering the questionnaire obtained higher scores (6.3 ± 2.0) than students who completed the questionnaire < 15 months (6.0 ± 1.8) and > 18 months (6.1 ± 2.0) after finishing their rotation. Respondents who intended to become obstetrician-gynecologists received the highest average score (6.7 ± 1.6) ; moreover, these respondents had a better understanding of HPV vaccination and cervical cancer screening compared with students in other intended specialties. No significant differences in average scores were observed within any subgroup (Table 3).

**Table 3:** Average scores by baseline characteristic

	Average score (SD)	p-value^a^
**Gender**		
Male	6.0 (2.1)	0.47
Female	6.2 (1.8)	
**Time between completing the department of obstetrics and gynecology rotation and answering the questionnaire**		
< 15 months	6.0 (1.8)	0.77
15–18 months	6.3 (2.0)	
> 18 months	6.1 (2.0)	
**Intended specialty**		
Obstetrics and gynecology	6.7 (1.6)	0.31
Others	6.1 (1.9)	

^a^Independent t-tests and one-way analyses of variance

## Discussion

Our study demonstrated that considerable knowledge gaps remain regarding HPV and cervical cancer prevention. The mean knowledge score was 6.12 ± 1.90, suggesting that only slightly more than half of the students scored higher than 50% on the questionnaire. Similar studies among medical students of comparable age have consistently highlighted poor knowledge levels regarding HPV and cervical cancer prevention [11–14]. For instance, a study in India assessing medical and paramedical student showed mean knowledge score was 5.19 ± 2.24 out of a total score of 17, indicating that most of the students scored less than 30% regarding cervical cancer and HPV vaccine [12]. Similarly, research in Poland found a mean knowledge score was 11.74 ± 2.51 points out of a maximum of 15 points regarding HPV, risk of cancer development, and vaccination [13]. Another survey conducted among medical students in Southwest China indicated that less than half of the students answered over 10 out of 22 questions on HPV-related knowledge [14].

A cross-sectional comparative study of medical students worldwide using Google Forms showed that American and European students possessed more knowledge about cervical cancer’s early signs, risk factors, and screening tests compared to their African and Asian counterparts [15]. These findings align with the observed low knowledge scores in our study, as well as those from studies in India and China [12, 14].

However, it’s important to note that the initial questionnaire underwent preliminary testing with first-year obstetrics and gynecology residents who specialize in cervical cancer prevention. Yet, we carefully considered their feedback and adjusted the questionnaire to suit the academic level of the targeted sixth-year medical students. These adaptations aimed to enhance the questionnaire’s clarity and relevance to the participants’ educational stage, thereby minimizing any complexity that might have influenced the survey outcomes.

Our study supported that gender, the time between completing the obstetrics and gynecology rotation and answering the questionnaire, and intended medical specialty were not significantly associated with the knowledge score.

Our survey provides valuable insights for shaping future educational programs targeting HPV and cervical cancer prevention. Addressing the knowledge gap in these areas through a well-structured curriculum is crucial. Previous studies have shown that educational interventions significantly enhance students’ understanding of HPV, highlighting the importance of educational programs for healthcare professionals, including those in medical universities [16].

Creating a robust educational framework is essential to equip medical students with comprehensive knowledge about cervical cancer and the HPV vaccine, enabling them to provide accurate information in their future clinical roles. Additionally, healthcare providers’ knowledge about HPV significantly influences their recommendations for vaccination [17]. Incorporating well-designed educational interventions into the academic curriculum can elevate students’ awareness of HPV-related diseases and prevention, guiding the development of more effective health promotion and education strategies [18].

As far as we know, our study stands as the initial detailed investigation into the understanding of HPV and cervical cancer prevention among Thai medical students. However, certain limitations must be acknowledged. Firstly, the data collection was limited to a single medical school, thus potentially lacking representation for all medical students in Thailand. Secondly, the study utilized a researcher-developed questionnaire due to the absence of a standardized validated questionnaire in the field of HPV and cervical cancer prevention education.

## Conclusion

Our survey underscores the insufficient comprehension among Thai medical students regarding HPV infection, vaccination, and cervical cancer screening protocols. Addressing this gap in the medical school curriculum by emphasizing education, communication, and raising awareness about these critical topics is imperative for successful cervical cancer prevention in the population.

## Data Availability

The data that support the findings of this study are available from the corresponding author upon request.
